# Whole Blood Transcriptome Analysis in Female Adolescents With Depression Accompanied by Nonsuicidal Self‐Injury and Suicide Attempt

**DOI:** 10.1155/da/6688695

**Published:** 2026-04-07

**Authors:** Zimo Zhou, Shuai Wang, Jiajun Yin, Zhenru Guo, Lianlian Yang, Xiaoshan Gao, Yu Xia, Yuanyuan Yang, Zhangyan Shan, Lin Tian

**Affiliations:** ^1^ The Affiliated Mental Health Center of Jiangnan University, Wuxi, Jiangsu, China; ^2^ School of Wuxi Medicine, Nanjing Medical University, Wuxi, Jiangsu, China, njmu.edu.cn

**Keywords:** adolescents, depression, nonsuicidal self-injury, RNA-seq, suicide attempt, transcriptome analysis

## Abstract

**Study Objective:**

Using RNA sequencing (RNA‐seq) on blood samples from depressed female adolescents with nonsuicidal self‐injury (NSSI) or suicide attempt (SA), this study explored links between peripheral blood gene expression and these behaviors.

**Methods:**

Female adolescents with major depressive disorder (MDD) who were of the same age as the subjects were enrolled in the study and divided into three groups: MDD, NSSI, and NSSI + SA. Whole blood samples were collected for RNA‐seq and bioinformatics analysis.

**Results:**

Multivariate linear regression analysis revealed that the NADH dehydrogenase iron–sulfur protein 7 (*NDUFS7*) and C–X–C motif chemokine ligand 10 (*CXCL10*) were important predictive factors distinguishing the MDD group from the NSSI + SA group. In weighted gene co‐expression network analysis (WGCNA), the blue module showed the greatest significance, with gene expression levels negatively correlated in the MDD group and NSSI group, and strongly positively correlated in the NSSI + SA group, consistent with differences in disease severity between self‐harm and suicide.

**Conclusion:**

This study revealed the whole‐blood transcriptomic features of female adolescent MDD patients with different behavioral phenotypes. It found that neuroimmune responses, along with core genes *NDUFS7* and *CXCL10*, are key in the development of NSSI and SA in adolescent females, and could be therapeutic targets.

**Trial Registration:**

ClinicalTrials.gov identifier: ChiCTR2500107586

## 1. Introduction

Major depressive disorder (MDD) has become one of the world’s most significant mental health stressors, as severe emotional and cognitive impairments disrupt patients’ affective responses, thought processes, and behavioral decision‐making mechanisms [1]. Globally, over 300 million people suffer from depressive symptoms, and the World Health Organization ranks it as the leading cause of disability [2]. Additional research indicates that the prevalence of depression has continued to rise in recent years [3]. Individuals with depression exhibit a higher risk of self‐harm compared to the general population, which in turn increases their risk of suicide [4, 5]. More critically, adolescents with moderate to severe depression face a suicide risk up to 30 times higher than that of the general population [6, 7].

Adolescent self‐harm and suicide are significant global public health issues [8, 9]. Adolescent self‐harm and suicidal behaviors are generally categorized into two distinct categories: nonsuicidal self‐injury (NSSI) and suicide attempt (SA). The latter constitutes the primary symptom of a suicidal disorder. SA is defined as self‐inflicted injury with the explicit intent to die [10]. The range of behaviors related to suicide is extensive and includes all forms, from suicidal ideation (SI) to SAs, ultimately resulting in suicide death. According to the World Health Organization, approximately 1 in 20 individuals who attempt suicide may ultimately succumb to their actions. The SA rate is approximately 17.76% [11]. Clinical research indicates that while suicide behaviors exhibit significant epidemiological differences across populations, their incidence is also influenced by sociodemographic factors, such as age, region, and gender [12–15]. NSSI is defined as deliberate and intentional self‐harm without suicidal intent. Common methods include cutting, hair pulling, head banging, biting, self‐mutilation, burns/scalds, and needle pricking [16, 17]. Despite the conceptual distinction between NSSI and SA, their inherent interconnectedness frequently results in their aggregation within the purview of self‐harm research. NSSI has been demonstrated to increase the risk of developing mental disorders and the likelihood of suicide resulting from self‐harm [18–22]. Furthermore, the frequency of NSSI can predict the probability of various suicide events occurring.

Depression serves as a significant precursor and comorbid factor for adolescent suicidal behavior, with its severity showing a significant positive correlation with suicide risk. A particularly salient finding is that adolescents who are female, have chronic medical or mental health conditions, experience persistent thoughts of death, possess a suicide plan, or have a family history of suicide exhibit significantly higher depression and suicide risk scores [23]. Moreover, emerging evidence indicates that lifestyle factors, such as nocturnal eating, inadequate sleep, and insufficient physical activity may also exacerbate depressive symptoms and suicide risk among adolescents by affecting emotional regulation and physiological rhythms [24]. Consequently, when assessing and intervening in adolescent self‐harm behaviors, it is imperative to systematically evaluate depressive mood, lifestyle patterns, and psychosocial risk factors in addition to clarifying the behavioral motivation (i.e., the presence of suicidal intent). This comprehensive approach provides a more thorough basis for early identification, risk stratification, and targeted interventions.

Current evidence implicates dysregulation of gene expression—particularly through transcriptomic and epigenetic pathways—as a pivotal neurobiological mechanism contributing to the development of these behavioral syndromes [25, 26]. Many studies have explored the search for key genes in NSSI and SA behaviors [27–34]. Despite previous genetic explorations of NSSI and SAs, the application of high‐throughput transcriptomic sequencing (RNA sequencing [RNA‐seq]) remains limited in this field. This technology provides an indispensable and unique tool for revealing the pathophysiological mechanisms underlying complex behavioral disorders [35, 36].

Recent clinical evidence has demonstrated that dysregulated peripheral inflammation—characterized by elevated expression of pro‐inflammatory cytokine/chemokine genes—shows a positive dose‐dependent association with the risk of suicidal thoughts and behaviors (STB) in adolescents. This suggests a potential neuroimmunological pathway underlying adolescent suicidality [37]. Due to their ease of acquisition, noninvasiveness, and dynamic response to disease states, such as inflammation and stress, whole blood samples have become an ideal vehicle for studying the association between gene expression and behavioral phenotypes [38, 39]. The use of whole‐blood transcriptomics is advantageous for studying the impact of mRNA levels present at a specific time point on nonsuicidal behavior in adolescents.

The mechanisms through which gender influences NSSI and SA remain largely unknown. Evident differences exist between males and females regarding self‐injury methods, stress coping, SAs, and mortality rates [40–43]. It is important to emphasize that gender profoundly influences the epidemiological characteristics and clinical phenotypes of NSSI and SA: female adolescents exhibit higher rates of NSSI, employ different self‐harm methods, and make more frequent SAs. However, transcriptomic studies specifically targeting this population remain scarce, representing a critical knowledge gap that urgently needs to be addressed.

This study investigated potential biological pathways associated with NSSI and SAs in female adolescents with depressive disorders using RNA‐seq based on whole blood samples. We collected three groups of samples: the SA group, the nonsuicidal self‐harm group, and the depression group—and subjected them to whole transcriptome sequencing. First, we identified differentially expressed messenger RNAs (mRNAs) and conducted a series of analyses based on these data. These analyses included biological process and pathway enrichment, protein–protein interaction (PPI) network, and weighted gene co‐expression network analysis (WGCNA). Finally, we identified promising candidate genes. These candidate genes may serve as supplementary clinical biomarkers. This study aims to reveal the specific biological pathways underlying NSSI and SA, screen for candidate biomarkers, assist in clinical diagnosis and risk stratification, and provide molecular targets for precision interventions.

## 2. Materials and Methods

### 2.1. Study Subjects

This is a single‐center study investigating changes in the whole‐blood cell transcriptomes of adolescents with depressive disorders who engage in NSSI and SA. The Ethics Committee of Wuxi Mental Health Center Hospital approved this study. All participants in this study were registered at the hospital and provided written informed consent. This study was conducted in accordance with the guidelines of the World Medical Association (Declaration of Helsinki). All study protocols were designed to minimize harm to participants and protect their rights.

We recorded questionnaire data for each group, including age, educational attainment, handedness, and mental disorders. We also recorded data from the following scales: Hamilton Rating Scale for Depression [44], Hamilton Rating Scale for Anxiety [45], Childhood Trauma Questionnaire–Short Form [46], Ottawa Self‐Injury Inventory [47], Beck’s Scale for SI, and 19‐Item Chen Internet Addiction Scale [48, 49]. Additionally, we recorded the detection rate, form, frequency, severity, and functional patterns of NSSI behaviors. Participants were divided into three groups: the NSSI group, the NSSI + SA group, and the MDD group.

The inclusion criteria for the adolescent depression disorder with NSSI behavior group are as follows: (1) Han Chinese ethnicity, right‐handedness, female gender, age between 12 and 25 years, and an educational level of at least elementary school; (2) no physical therapy, including electroconvulsive therapy without convulsions, within 3 months prior to enrollment; (3) meeting the diagnostic criteria for depression according to the DSM‐5; and (4) meeting the DSM‐5 definition of NSSI, which is defined as intentional self‐harm without suicidal intent, and having engaged in NSSI behavior at least five times within 12 months prior to biological sample collection.

The following inclusion criteria have been established for the adolescent depression disorder with suicidal behavior group: (1) subjects must be of Han Chinese ethnicity, right‐handed, female, aged 12–25 years, and with an educational level of elementary school or above; (2) subjects may not have undergone physical therapy, including nonconvulsive electroconvulsive therapy, within 3 months prior to enrollment; (3) subjects must meet the diagnostic criteria for depression according to DSM‐5. Fourth, participants who meet the DSM‐5 definition of suicidal behavior (defined as intentional self‐harm resulting in injury to bodily tissue with suicidal intent) must have had at least one episode of suicidal behavior within the 24 months prior to the collection of biological samples.

The following inclusion criteria were established for the group of adolescents with depressive disorders without NSSI and suicidal behavior: (1) Han Chinese ethnicity, right‐handedness, female, aged 12–25 years, and with an educational level of elementary school or above [50]; (2) no physical therapy, including nonconvulsive electroconvulsive therapy, within 3 months prior to enrollment; (3) meets the DSM‐5 diagnostic criteria for depression; and (4) according to the DSM‐5 definition of NSSI behavior (i.e., the subject exhibited no instances of intentional self‐harm without suicidal intent within 12 months prior to the collection of biological samples. According to the DSM‐5 definition of suicidal behavior (i.e., intentional self‐harm with suicidal intent), no such instances occurred within 24 months prior to the collection of biological samples.

**Figure 1:**
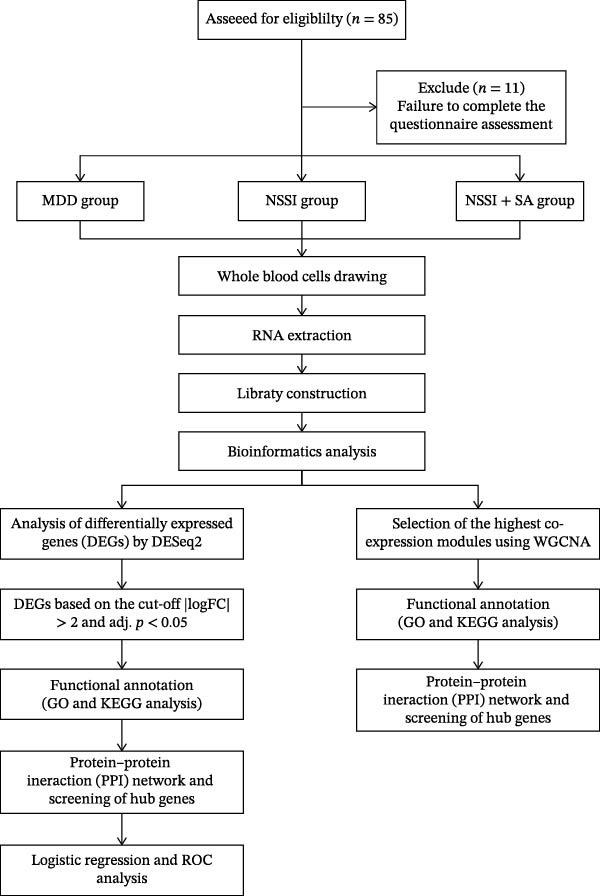
Schematic flow diagram of the study. DEGs, differentially expressed genes; GO, Gene Ontology; KEGG, Kyoto Encyclopedia of Genes and Genomes; WGCNA, weighted gene co‐expression network analysis; PPI, protein–protein interaction.

Exclusion criteria: (1) current or past history of neurological disorders or other severe physical illnesses; (2) patients with a history of epilepsy or severe head trauma; (3) pregnant or breastfeeding women, or those planning to become pregnant; and (4) Age under 12 years or over 25 years;

A total of 85 participants were screened, of whom 11 were excluded due to their refusal to complete the questionnaire. Finally, whole blood samples were collected from each group of participants, with 36 participants in the group with a history of depressive disorder accompanied by NSSI and SAs, defined as the NSSI + SA group. The present study’s sample comprised 24 participants diagnosed with depressive disorders and a history of NSSI. Fourteen participants diagnosed with depressive disorders without NSSI or SAs were defined as the MDD group. Subsequently, the protocol was continued with the extraction of RNA, the construction of libraries, and bioinformatics analysis, as illustrated in the Figure 1.

### 2.2. Blood Sample Collection

PAXgene blood collection tubes have been demonstrated to protect RNA, thereby ensuring minimal RNA degradation during extended storage periods. A total of 2.5 mm of whole blood should be collected using PAXgene tubes. The tubes should be stored upright at room temperature (18–25°C) for a minimum of 2 h. Thereafter, the tubes should be transferred to a −80°C freezer for long‐term storage [51].

### 2.3. RNA Qualification and Library Construction

In our study, according to the manufacturer’s instructions, 1 μg total RNA was used for following library preparation. The poly(A) mRNA isolation was performed using Oligo(dT) beads. The mRNA fragmentation was performed using divalent cations and high temperature. Priming was performed using random primers. First‐strand cDNA and the second‐strand cDNA were synthesized. The purified double‐stranded cDNA was then treated to repair both ends and add a dA‐tailing in one reaction, followed by a T–A ligation to add adaptors to both ends. Size selection of Adaptor‐ligated DNA was then performed using DNA clean beads. Each sample was then amplified by PCR using P5 and P7 primers and the PCR products were validated. Then libraries with different indexes were multiplexed and loaded on an Illumina Novaseq instrument for sequencing using a 2 × 150 paired‐end (PE) configuration according to manufacturer’s instructions.

### 2.4. Sample Processing and Bioinformatics Analysis

The RNA was extracted from whole blood samples, and reverse transcription was subsequently performed to construct a gene expression library. To that end, we employed bioinformatics analysis to examine the differentially expressed genes (DEGs) between the groups, the pathways that they were associated with, and the WGCNA analysis. Finally, potential biomarkers and pathways associated with the MDD–NSSI + SA and NSSI–NSSI + SA groups were identified at the level of whole blood cells. The subsequent sections provide detailed information regarding sample processing and bioinformatics analysis.

### 2.5. DEG Identification

Differential expression analysis used the DESeq2 Bioconductor package, a model based on the negative binomial distribution. The estimates of dispersion and logarithmic fold changes incorporate data‐driven prior distributions, *p*‐adj of genes were setted ≤ 0.05 to detect differential expressed ones. A gene was designated as an upregulated DEG if the *p*‐adj value was less than 0.05 and the log_2_ (fold change) was greater than 2. A gene was designated as a downregulated DEG if the *p*‐value was less than 0.05 and the log_2_ (fold change) was less than −2. The identification of between‐group DEGs was conducted, and their corresponding data was presented in the form of volcano maps utilizing R software [52].

### 2.6. Pathway Enrichment Analysis

GOSeq (v1.34.1) was used identifying Gene Ontology (GO) terms that annotate a list of enriched genes with a significant *p*‐adj less or equal than 0.05, and top GO was used to plot DAG. Kyoto Encyclopedia of Genes and Genomes (KEGG) is a collection of databases dealing with genomes, biological pathways, diseases, drugs, and chemical substances (http://en.wikipedia.org/wiki/KEGG) [53, 54].

### 2.7. PPI Network

The STRING database (http://string-db.org/) is a tool that is used for PPI analysis of DEGs that have undergone enrichment screening. The Cytoscape software was utilized for the purpose of visualizing the PPI analysis. In the Cytoscape platform, each node denotes a protein or gene, with lines denoting molecular interactions between them. The number of lines connecting a protein directly corresponds to the number of interactions it has. Modules in Cytoscape can be utilized to identify the most closely related DEGs in the entire PPI network and visualize them.

### 2.8. WGCNA

We performed a WGCNA using the WGCNA package in R, which defines gene networks based on the correlations between genes [55]. In these networks, genes are represented as nodes, and edges represent the connections between them. The networks are then divided into modules of gene clusters with highly coordinated expression. The gene expression profile of each module is summarized by calculating the module eigengene, which is defined as the first principal component of the module’s expression matrix. Then, a module membership measure is assigned to each gene. To identify biologically meaningful modules, we assigned a gene significance measure for each trait to each gene by calculating the absolute correlation between the trait and the expression profile. Next, we calculated the module‐trait significance by correlating the module membership value with the gene significance value. We considered the association significant if the *p*‐value exceeded the Bonferroni correction (*p* ≤ 0.05) when testing multiple modules.

### 2.9. Statistical Analysis

The statistical analysis of the data was conducted using SPSS 27.0. Quantitative data that is normally distributed is described as mean ± standard deviation (*x* ± *s*). Multiple groups were compared using one‐way analysis of variance (ANOVA), and LSD *t*‐test was used for multiple pair‐wise comparisons between groups. Quantitative data that was not normally distributed was expressed as *M* (P25–P75). Multiple groups were compared using the Kruskal–Wallis *H* rank sum test. Binary logistic regression analysis was utilized to identify the influencing factors, and the variance inflation factor (VIF) was employed to test for multicollinearity in the regression model. Statistically, a *p*‐value less than 0.05 was considered to be significant.

## 3. Results

### 3.1. Baseline Characteristics

In the inclusion and analysis process for depression, sample processing and data generation are first conducted. These processes encompass whole blood cell collection, RNA extraction, and library construction. Subsequently, DEGs are identified through bioinformatics analysis and screened based on statistical thresholds. Subsequently, functional annotation and pathway analysis are performed on the target gene sets. Finally, key modules and hub genes are subjected to further screening via protein interaction networks, thereby advancing from data to biological interpretation (Figure 1). The present study compared baseline demographic and clinical characteristics among three groups: the presence of MDD, NSSI + SA, and NSSI was identified. The following table systematically presents the distribution and intergroup comparisons across the three groups regarding age, years of education, severity of depression and anxiety, suicide‐related indicators, internet addiction, and childhood trauma. The objective of this comparison is to elucidate the clinical heterogeneity among the groups, thereby providing essential demographic and clinical context for subsequent exploration of mechanisms and interpretation of outcomes (Table 1). There were no significant differences between the three groups in terms of age (*h* = 0.73, *p* = 0.694) and years of education (*h* = 4.3, *p* = 0.116). Intergroup comparisons revealed significant differences in 14‐item Hamilton Rating Scale for Anxiety total scores (*h* = 14.54, *p* < 0.001), 17‐item Hamilton Rating Scale for Depression total scores (*h* = 11.65, *p* = 0.003), suicide ideation scores (in the past 1 week: *z* = −3.67, *p* < 0.001; during the hardest time: *z* = −4.29, *p* < 0.001), and suicide risk score (in the past 1 week: *t* = ‐3.05, *p* = 0.002; during the hardest time: *t* = −3.02, *p* < 0.001), emotional neglect in the Childhood Trauma Questionnaire–Short Form (*h* = 10.49, *p* = 0.005), emotional abuse (*h* = 18.17, *p*  < 0.001), physical abuse (*h* = 10.88, *p* = 0.004), withdrawal symptoms of internet addiction in the 19‐item Chen Internet Addiction Scale (*h* = 6.97, *p* = 0.031), tolerance symptoms of internet addiction (*h* = 10.86, *p* = 0.004), and interpersonal and health‐related problems of internet addiction (*h* = 7.12, *p* = 0.028).

**Table 1:** Baseline characteristics of participants in the three groups.

Variables	MDD (*n* = 14)	NSSISA (*n* = 36)	NSSI (*n* = 24)	Statistical value	*p*‐Value
Age (years)	16.50 (14.00, 18.00)	15.00 (14.00, 17.00)	15.00 (14.00, 17.75)	0.73	0.694^a^
Education (years)	9.50 (8.75, 11.00)	9.00 (8.00, 9.75)	9.00 (8.00, 13.50)	4.3	0.116^a^
HAMA‐14	18.00 (13.25, 26.25)	27.50 (23.25, 35.00)	24.00 (15.75, 27.50)	14.54	0.001 ^∗∗∗^^a^
HAMD‐17	18.00 (14.75, 22.50)	23.50 (21.00, 26.75)	20.00 (16.25, 27.25)	11.65	0.003 ^∗∗^^a^
Suicide ideation					
In the past 1 week	—	11.00 (10.00, 13.00)	9.00 (6.00, 10.75)	−3.67	0.001 ^∗∗∗^^c^
During the hardest time	—	52.44 ± 18.05	29.29 ± 16.68	−4.29	0.001 ^∗∗∗^^b^
Suicide risk					
In the past 1 week	—	15.00 (14.00, 15.00)	13.00 (11.25, 15.00)	−3.05	0.002 ^∗∗^^c^
During the hardest time	—	73.98 ± 12.26	61.99 ± 15.45	−3.02	0.001 ^∗∗∗^^b^
CIAS‐19					
Sym‐C	7.00 (7.00, 9.00)	8.00 (7.00, 9.00)	7.00 (5.25, 8.75)	3.18	0.203^a^
Sym‐W	8.00 (6.75, 8.00)	8.00 (7.00, 9.00)	7.00 (6.00, 8.00)	6.97	0.031 ^∗^^a^
Sym‐T	9.00 (8.75, 10.00)	11.00 (10.00, 13.00)	9.00 (8.00, 11.75)	10.86	0.004 ^∗∗^^a^
RP‐IH	11.00 (10.00, 13.00)	12.00 (10.25, 13.00)	10.00 (8.00, 11.75)	7.12	0.028 ^∗^^a^
RP‐TM	8.86 ± 1.79	10.22 ± 2.37	8.92 ± 3.08	2.54	0.086^d^
CTQ‐SF					
Sexual abuse	5.00 (5.00, 5.00)	5.00 (5.00, 5.00)	5.00 (5.00, 5.75)	0.84	0.657^a^
Physical abuse	5.00 (5.00, 6.00)	7.00 (5.00, 11.75)	6.00 (5.00, 10.00)	10.88	0.004 ^∗∗^^a^
Emotional abuse	7.00 (6.50, 10.00)	14.00 (11.00, 18.00)	12.00 (10.50, 16.00)	18.17	0.001 ^∗∗∗^^a^
Physical neglect	9.00 (7.00, 10.25)	10.00 (8.00, 11.00)	8.00 (7.00, 9.75)	4.21	0.122^a^
Emotional neglect	17.00 (11.25, 18.25)	17.00 (13.25, 21.00)	13.00 (10.25, 16.00)	10.49	0.005 ^∗∗^^a^

*Note*: HAMA‐14, 14‐item Hamilton Rating Scale for Anxiety; HAMD‐17, 17‐item Hamilton Rating Scale for Depression; MDD, depressed female adolescents without nonsuicidal self‐injury and suicide attempts; NSSI, depressed female adolescents with nonsuicidal self‐injury alone; NSSISA, depressed female adolescents with nonsuicidal self‐injury and suicide attempts; RP‐IH, interpersonal and health‐related problems of internet addiction; RP‐TM, time management problems; Sym‐C, compulsive use of internet; Sym‐T, tolerance symptoms of internet addiction; Sym‐W, withdrawal symptoms of internet addiction.

Abbreviations: BSSI, Beck’s Scale for Suicidal Ideation; CIAS‐19, 19‐item Chen Internet Addiction Scale; CTQ‐SF, Childhood Trauma Questionnaire–Short Form.

^a^Three‐sample Kruskal–Wallis test, median (lower quartile, upper quartile).

^b^Two independent‐samples *t*‐test, mean (standard deviation).

^c^Two‐sample Mann–Whitney *U* test, median (lower quartile, upper quartile).

^d^Chi‐square test.

^∗^ *p* < 0.05.

^∗∗^ *p* < 0.01.

^∗∗∗^ *p* < 0.001.

### 3.2. Identification of DEGs

RNA was extracted from whole blood samples, and a cDNA library was constructed to analyze the expression differences of tens of thousands of genes between the two groups. The present study delineates a differential gene expression analysis workflow and core findings predicated on whole‐blood RNA‐seq data. The research first obtained whole‐transcriptome expression data through RNA extraction and library construction, then systematically compared gene expression profiles between the MDD–NSSISA and NSSI–NSSISA groups. The analysis identified genes that were significantly upregulated and downregulated in each group, visually represented via volcano plots. The magnitude of expression changes was further reflected by the absolute value of log_2_ (FC), suggesting that genes with greater expression differences may hold greater importance in phenotype‐related biological functions (Figure 2). In the MDD–NSSISA group, the NSSISA subgroup exhibited 1276 DEGs that were upregulated (*p* < 0.05, log_2_ [FC] > 2) and 245 DEGs that were downregulated (*p* < 0.05, log_2_ [FC] < −2) (Figure 2A). In the NSSI–NSSISA group, the NSSISA subgroup exhibited 1658 upregulated DEGs (*p* < 0.05, log_2_ [fold change] >2) and 408 downregulated DEGs (*p* < 0.05, log_2_ [fold change] < −2) (Figure 2B). However, our attempts to identify DEG clusters between the MDD–NSSI groups were unsuccessful. These findings reveal transcriptome heterogeneity across groups at the genome‐wide level, providing a critical gene list and trend analysis for subsequent functional enrichment and mechanism studies.

Figure 2Screening for DEGs. (A) Volcano plot of DEGs in MDD–NSSI + SA. (B) Volcano plot of DEGs in NSSI–NSSI + SA. DEGs, differentially expressed genes.

**Figure:**
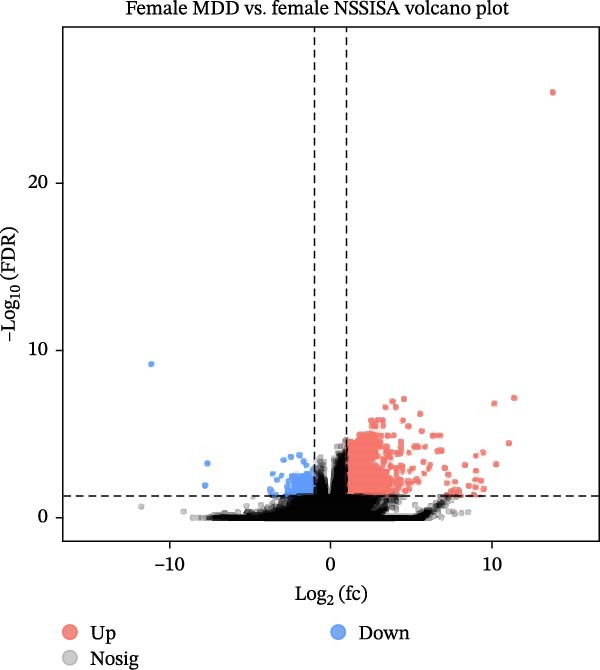
(A)

**Figure:**
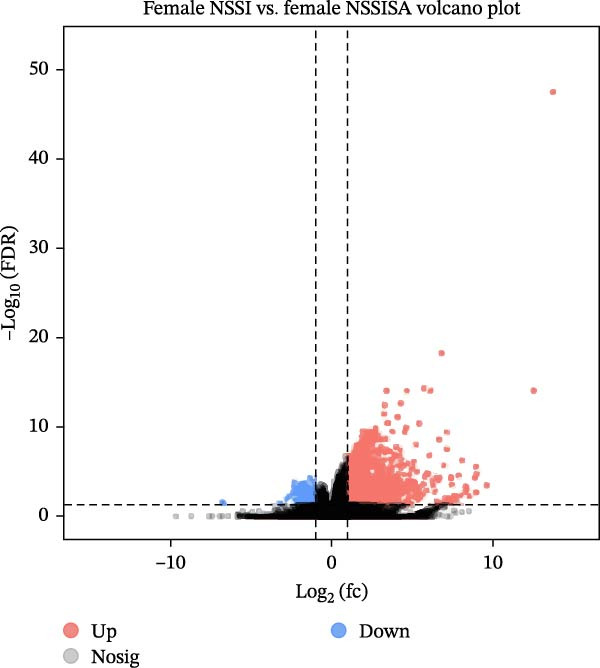
(B)

### 3.3. Pathway Enrichment Analysis

To further explore the pathways associated with the pathogenesis of MDD–NSSI + SA and NSSI–NSSI + SA in female adolescents, we performed pathway enrichment analysis on 1521 DEGs in the MDD–NSSI + SA group and 2066 DEGs in the NSSI–NSSI + SA group. GO and KEGG pathway enrichment analysis were then employed to group discrete gene expression changes into pathways and functional modules with clear biological significance. The objective of this analysis was to elucidate the underlying common biological processes and signaling networks that underpin phenotypic differences. Subsequently, functional annotation was performed on the DEGs obtained from the MDD–NSSISA and NSSI–NSSISA comparisons, respectively. The enrichment results were then represented in the form of bubble plots, where the size of the dots corresponded to the number of enriched genes and the color signified the enrichment significance (Figure 3). In the MDD–NSSI + SA group, DEGs that were enriched in GO analysis were found to be associated with ribosome‐related pathways, including ribosomal subunit, cytosolic ribosome, structural constituent of ribosome, ribosome, mitochondrial protein–containing complex, and extracellular space (Figure 3A,B). KEGG analysis revealed that DEGs were enriched in pathways including ribosome, oxidative phosphorylation, thermogenesis, pathways of neurodegeneration—multiple diseases, and rheumatoid arthritis. In the NSSI–NSSI + SA group, DEGs in the GO analysis were found to be enriched in terms of the cytosolic ribosome, ribosomal subunit, structural constituent of ribosome, ribosome, cytosolic large ribosomal subunit, and cytosolic small ribosomal subunit. A subsequent investigation employing the KEGG database revealed that DEGs were enriched in the ribosome and oxidative phosphorylation pathways (Figure 3C,D).

Figure 3Screening for GO and KEGG. (A) The GO outcomes are displayed with a bubble plot in the MDD–NSSI + SA group. (B) A bubble plot was constructed to illustrate the KEGG outcomes in the MDD–NSSI + SA group. (C) The GO outcomes are displayed with a bubble plot in the NSSI–NSSI + SA group. (D) A bubble plot was constructed to illustrate the KEGG outcomes in the NSSI–NSSI + SA group.

**Figure:**
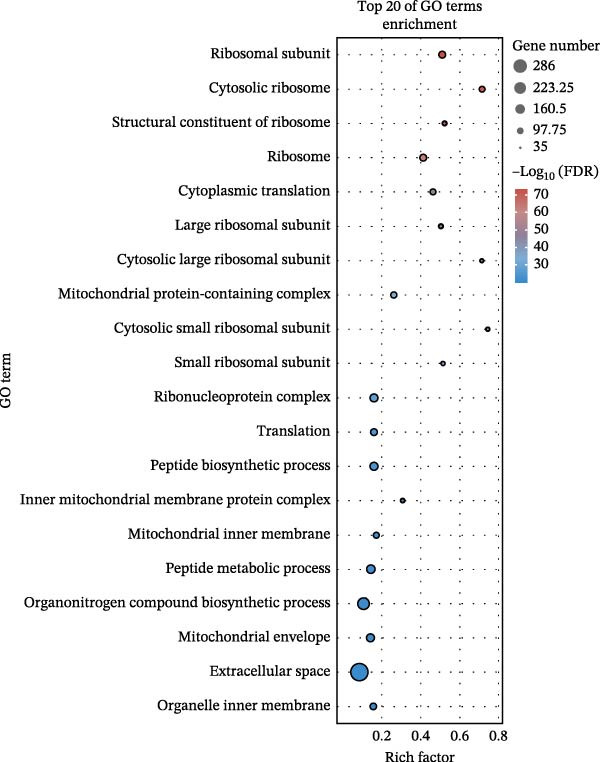
(A)

**Figure:**
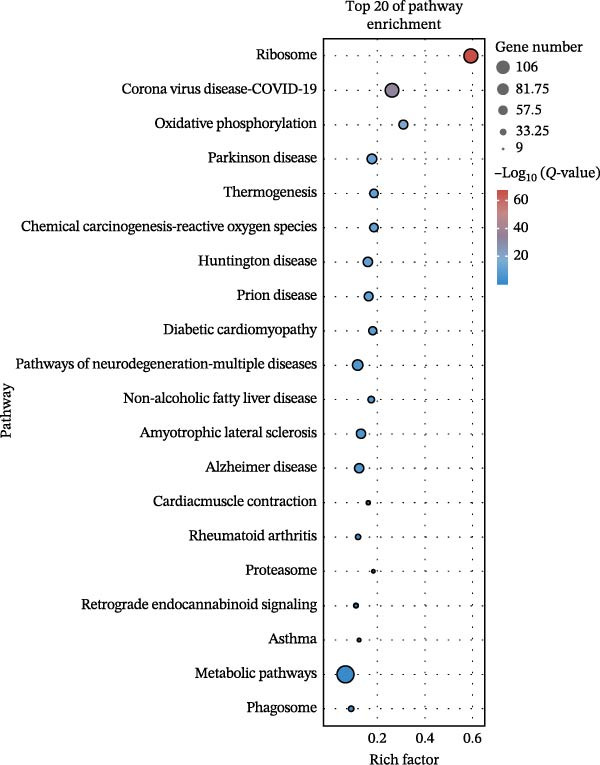
(B)

**Figure:**
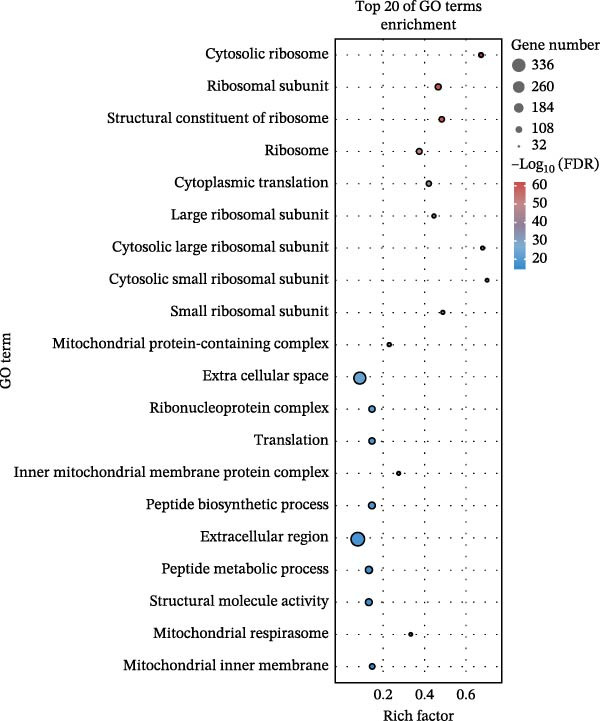
(C)

**Figure:**
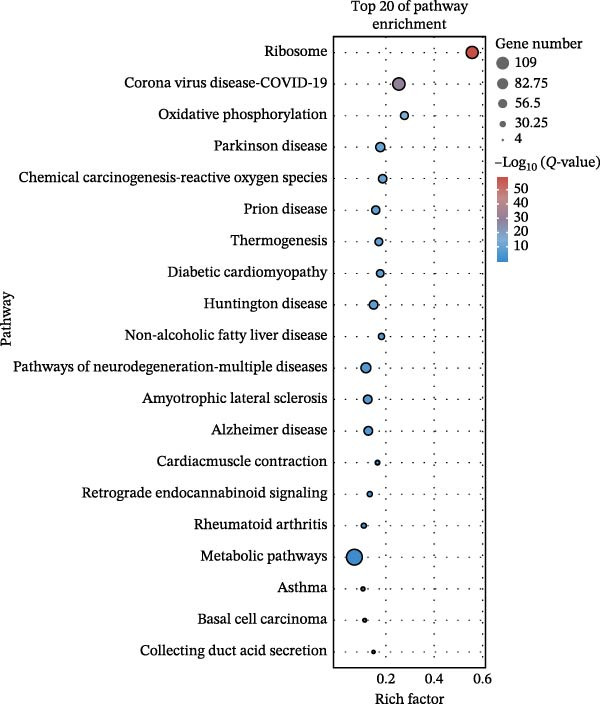
(D)

### 3.4. PPI Network and Hub Gene Selection

The PPI network describes the relationships between genes and identifies those most closely related to others (central genes). We used the STRING protein interaction database (http://string-db.org/) to analyze the protein interaction network of DEGs, primarily using the interaction relationships in the database. The STRING database is a system for searching known PPIs and predicting PPIs. These interactions include direct physical interactions and indirect functional relationships between proteins. The analysis demonstrated the complete workflow from differential expression genes to the screening of key hub genes through PPI network analysis. In accordance with the potential functional modules indicated by the preceding KEGG/GO enrichment analysis, the PPI network of DEGs was subsequently constructed using the STRING database to identify key genes occupying central regulatory positions within the network (Figure 4). The analysis began with the extraction of known and predicted interactions for the target gene set from the STRING database (Figure 4B,D). This was followed by network visualization and topological analysis using Cytoscape software. By calculating the degree of connectivity for each node in the network, the study identified the top 20 genes with the highest connectivity as key hub genes (Figure 4A,C).

Figure 4Screening hub genes by PPI network. (A) Hub genes in the MDD‐NSSI + SA group were analyzed using Cytoscape software. (B) Hub genes in the MDD‐NSSI + SA group were identified through screening of protein–protein interaction networks. (C) Hub genes in the NSSI‐NSSI + SA group were analyzed using Cytoscape software. (D) Hub genes in the NSSI‐NSSI + SA group were identified through screening of protein–protein interaction networks.

**Figure:**
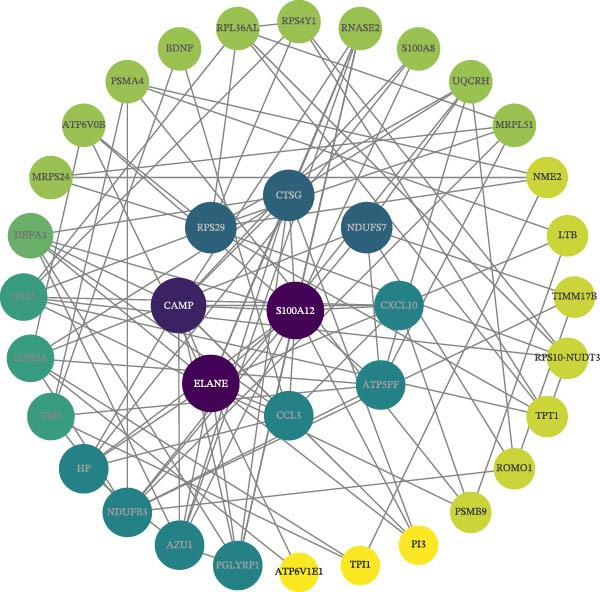
(A)

**Figure:**
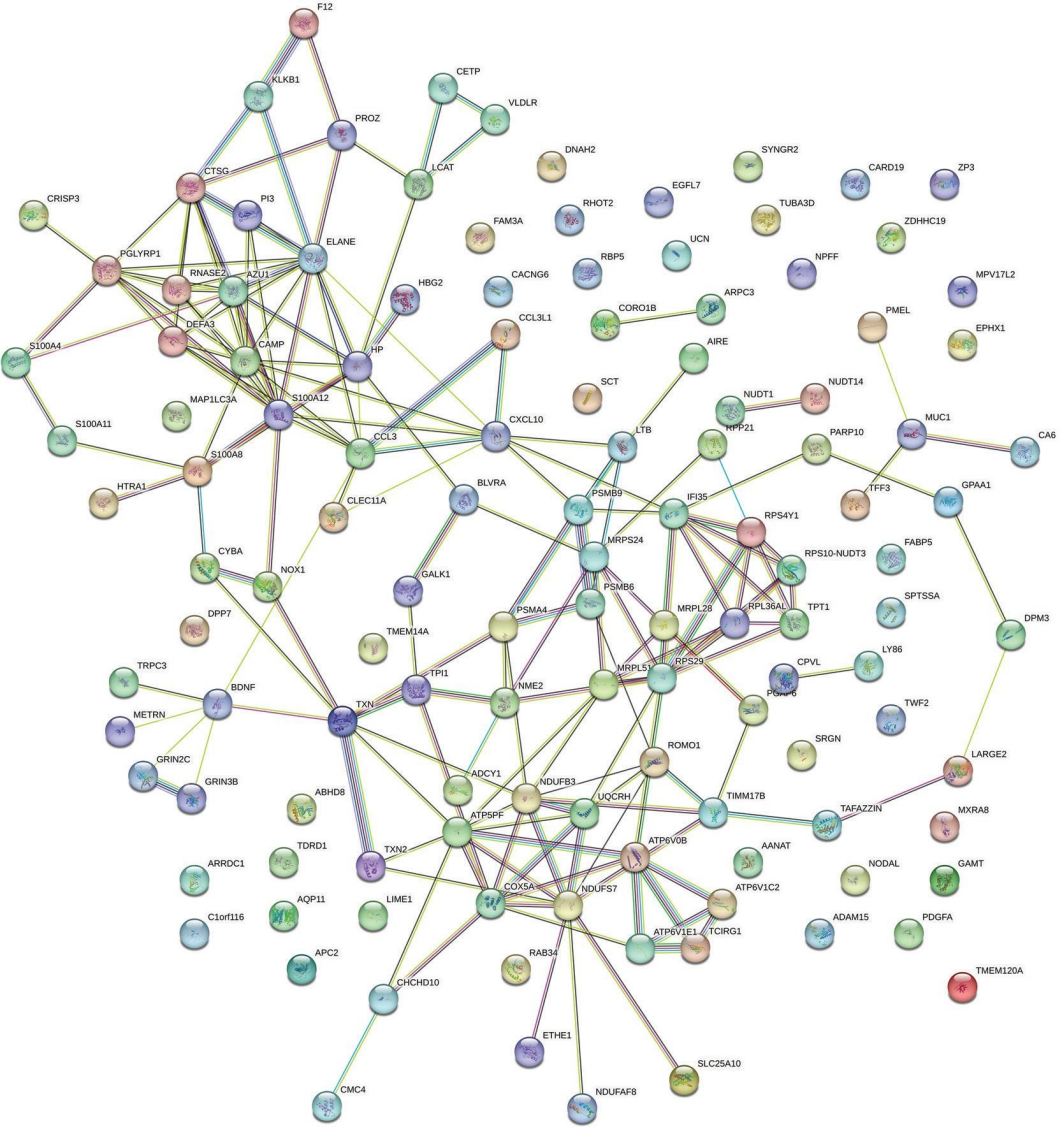
(B)

**Figure:**
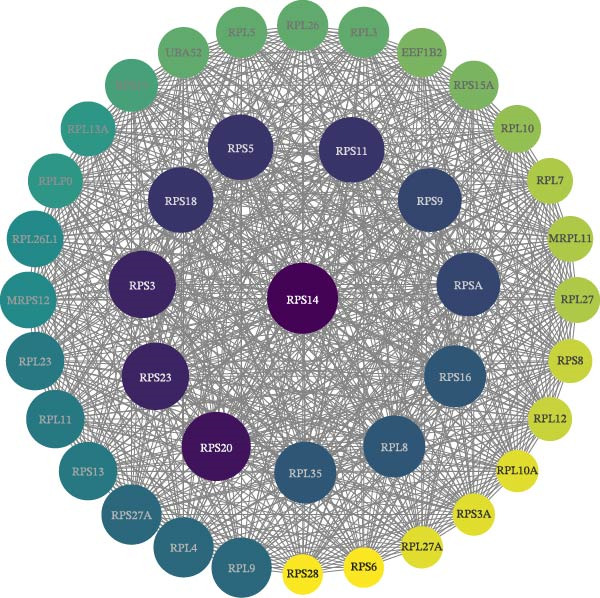
(C)

**Figure:**
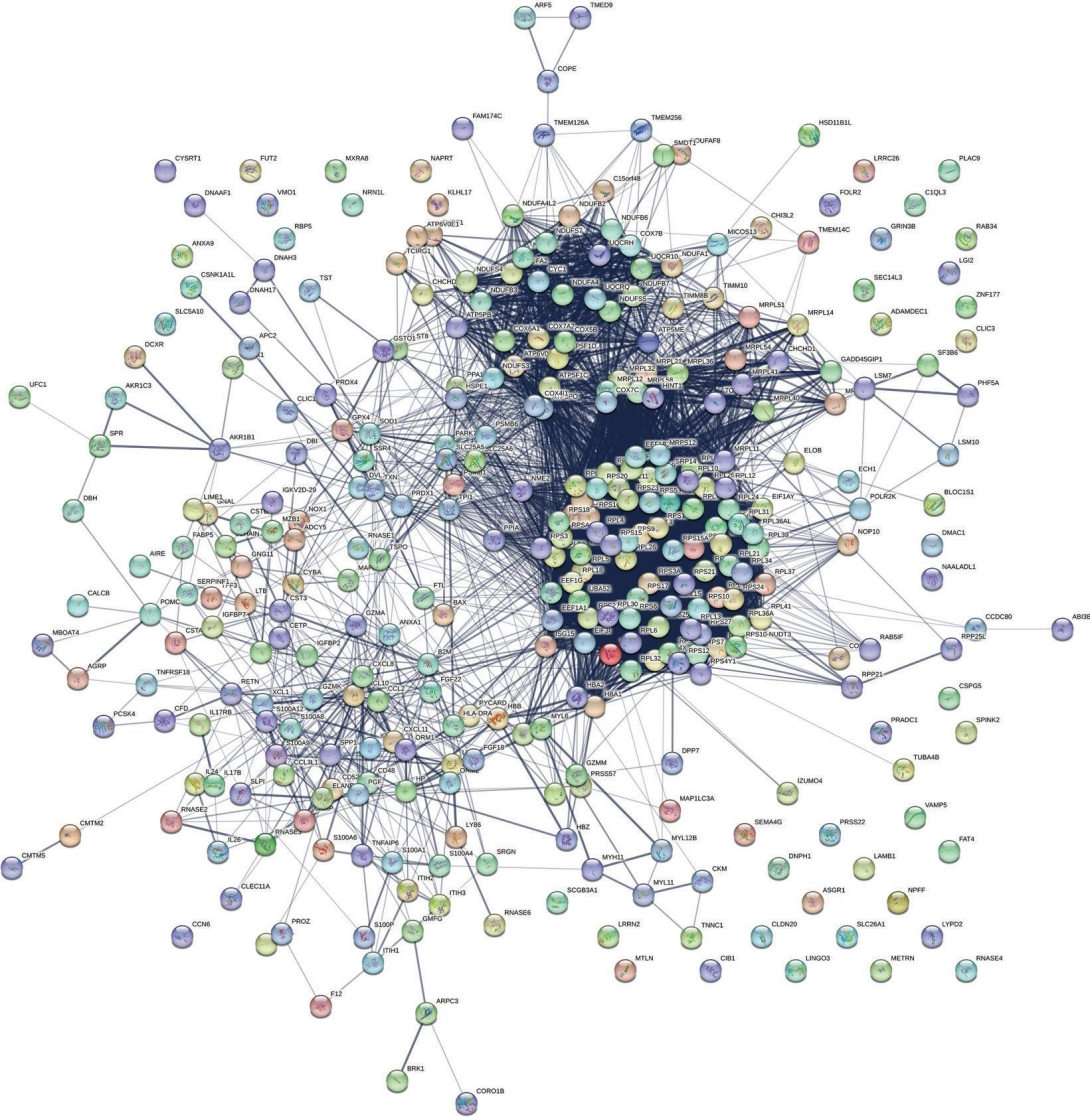
(D)

### 3.5. Association Between the Group and the Genes Was Evaluated With Multiple Linear Regression Analyses

The 20 key genes screened based on the PPI network were subjected to binary logistic regression analysis in the MDD–NSSI + SA group and the NSSI–NSSI + SA group. In the MDD–NSSISA group, the most critical hub genes were NADH dehydrogenase iron–sulfur protein 7 (*NDUFS7*) and C–X–C motif chemokine ligand 10 (*CXCL10*) (Figure 4A,B). In the NSSI–NSSI + SA group, the most critical hub gene was the gene encoding ribosomal protein L8 (*RPL8*). In the MDD–NSSI + SA group, the regression coefficient value for the analyte, denoted as *CXCL10* was 3.578, and it demonstrated significance at the 0.05 level (*z* = 2.572, *p* = 0.010), thereby indicating that the analyte has a significant positive influence on MDD–NSSI + SA. The odds ratio (OR) value was determined to be 35.815. The regression coefficient value for *NDUFS7* was 5.167, and it demonstrated significance at the 0.05 level (*z* = 2.544, *p* = 0.011), thereby indicating that *NDUFS7* exerts a significant positive influence on MDD–NSSI + SA. The OR value was 175.329. In the NSSI–NSSI + SA group, the regression coefficient value for *RPL8* is 1.228, and it shows significance at the 0.01 level (*z* = 2.885, *p* = 0.004), indicating that *RPL8* has a significant positive influence on NSSI–NSSI + SA. The OR value was 3.413 (Table 2).

**Table 2:** Binary logit regression analysis results.

Variable	Regression coefficient	Standard error	*z*‐value	Wald *χ* ^2^	*p*‐Value	Odds ratio (OR)	95% CI for OR
CXCL10	3.578	1.392	2.572	6.613	0.01 ^∗^	35.815	2.342–547.696
NDUFS7	5.167	2.031	2.544	6.473	0.011 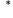	175.329	3.276–9384.254
RPL8	1.228	0.426	2.885	8.321	0.004 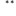	3.413	1.482–7.860

*Note*: NDUFS7, NADH dehydrogenase iron–sulfur protein 7.

Abbreviations: CXCL10, chemokine (C–X–C motif) ligand 10; RPL8, ribosomal protein L8.

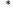
 *p* < 0.05.

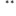
 *p* < 0.01.

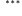
 *p* < 0.001.

### 3.6. ROC Curve Analysis

To explore the diagnostic efficacy of the three hub genes, we implemented a ROC curve analysis in which hub genes with an AUC value > 0.7 were used as diagnostic markers. In the female MDD–NSSI + SA group, the AUC values were 0.825 for *NDUFS7* (95% CI: 0.707–0.944), 0.840 for *CXCL10* (95% CI: 0.724–0.957) (Figure 5A,B). In the female NSSI–NSSI + SA group, the AUC values were 0.564 for *RPL8* (95% CI: 0.417–0.710) (Figure 5C).

Figure 5ROC curve analysis. (A) Hub genes in the MDD–NSSI + SA group were analyzed using ROC curves. (B) Hub genes in the NSSI–NSSI + SA group were analyzed using ROC curves.

**Figure:**
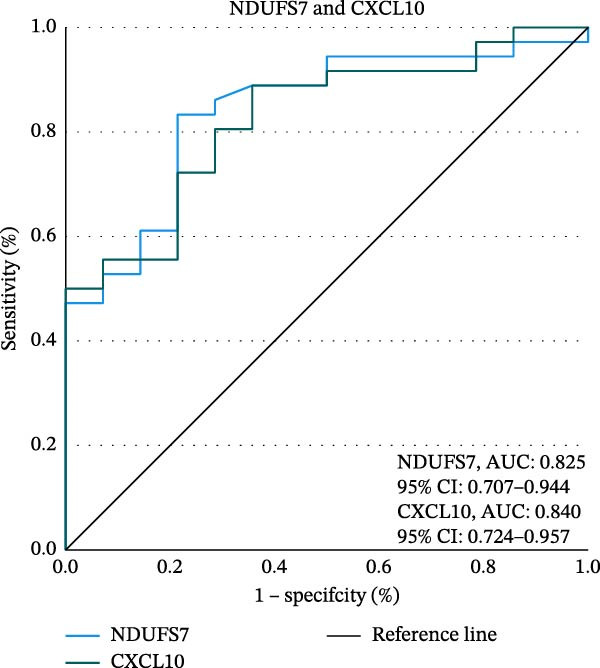
(A)

**Figure:**
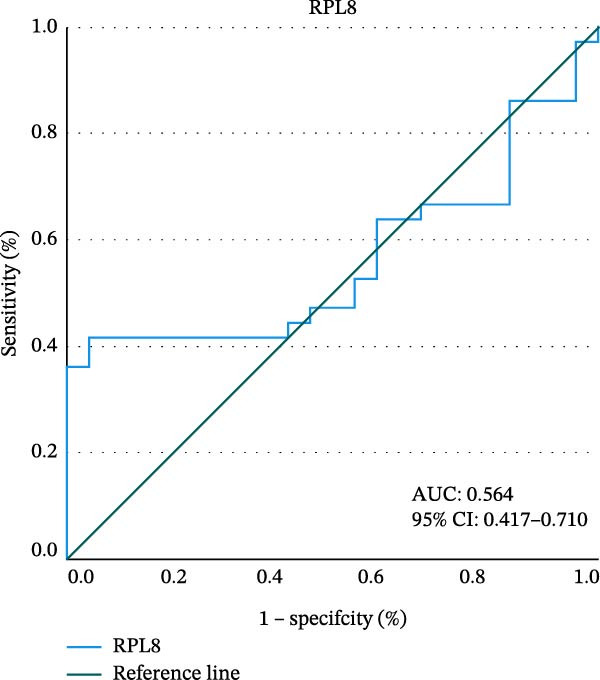
(B)

### 3.7. WGCNA

The present study employed WGCNA to construct gene expression modules and further explore the associations between key modules and target clinical features. Initially, a clustering analysis was conducted on the samples (Figure 6A). As the soft threshold power (*β* value) increased, the network’s scale‐free topological fit index exhibited a gradual rise. At a value of *β* = 10, the *R*‐squared value reached 0.85, thereby meeting the empirical standard of typically exceeding 0.8. This finding suggests that the network demonstrated notable scale‐free properties at this juncture, whereby a limited number of hub genes were interconnected with a substantial number of nodes. This observation is consistent with the topological configuration of numerous biological networks. The right panel of Figure 1 illustrates that the average connectivity, defined as the average number of connections per gene, experiences a gradual decrease as the parameter *β* increases. The selection of *β* = 10 guarantees that the network complies with the scale‐free property while circumventing the undesirable consequence of excessive filtering of authentic biological associations, which would otherwise result from the implementation of excessively elevated thresholds. This approach effectively eliminates random noise and preserves gene co‐expression relationships that are of significant biological importance (Figure 6B,C). Genes within the same module may exhibit similar expression patterns and participate in the same biological processes. The modules were associated with the presence of depression, NSSI, and SAs. We calculated the importance of genes and gene module members for poly‐morbidity. We identified multiple gene co‐expression modules that were significantly associated with female self‐harm behavior and its severity, as well as regulatory factors. The MEblue module exhibited the most significant bidirectional association pattern (Figure 6D). It showed a significant negative correlation with MDD (*r* = −0.26, *p* = 0.03), suggesting that low expression of genes in this module may be associated with weaker regulatory factors related to the risk of female self‐harm behavior. Interestingly, the MEblue module showed a strong positive correlation with NSSI + SA (*r* = 0.55, *p* =  1e – 06), suggesting that elevated gene expression levels in this module may be an important biomarker or driver of increased NSSI severity. This was particularly evident in the female suicide group, where higher MEblue module gene levels were associated with more severe clinical manifestations. Enrichment analysis of the module’s genes revealed GO enrichment involving genes associated with innate immunity, antiviral responses, phagocytosis, and granule release mechanisms. This suggests that these cells may be neutrophils, natural killer (NK) cells, or other immune cells involved in antiviral responses (Figure 6E‐G). These genes may also be actively expressed during infection, inflammation, or immune responses, and KEGG pathways collectively reflect high immune activity and infection responses (Figure 6H). PPI analysis revealed that the core gene ISG15 belongs to the interferon‐stimulated gene (ISG) family. ISGs have functions including antiviral defense, immune regulation, and post‐translational modification. IFI genes, as core executors of the interferon response, directly participate in regulating inflammatory responses and respond to noninfectious stress, such as oxidative and endoplasmic reticulum stress (Figure 6I,J).

Figure 6(A) Clustering dendrogram of samples with trait heatmap. (B) Analysis of network topology for various soft‐thresholding powers. The left panel shows the scale‐free fit index (*y*‐axis) as a function of the soft‐thresholding power (*x*‐axis). The right panel displays the mean connectivity (degree, *y*‐axis) as a function of the soft‐thresholding power (*x*‐axis). (C) Clustering dendrogram of genes, with dissimilarity based on topological overlap, together with assigned module colors. (D) Module–trait associations: each row corresponds to a module eigengene and each column to a trait. Each cell contains the corresponding correlation and *p*‐value. The table is color‐coded by correlation according to the color legend. Distribution of average gene significance and error in modules associated with female adolescent depression disorder patients. (E–H) Screening for GO and KEGG. (I, J) Screening hub genes by PPI network.

**Figure:**
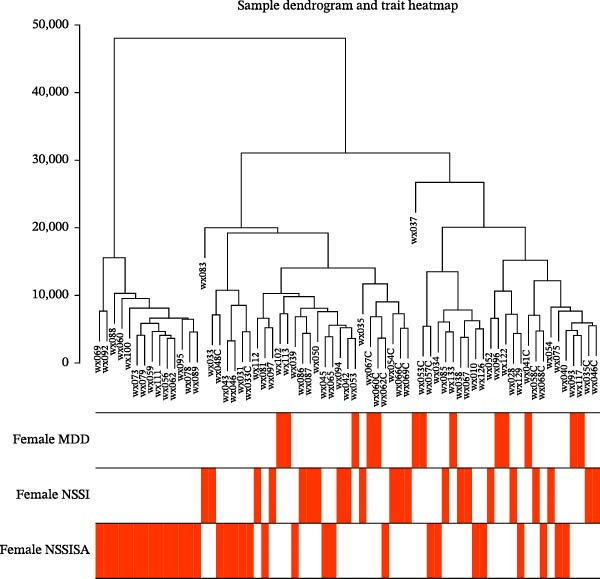
(A)

**Figure:**
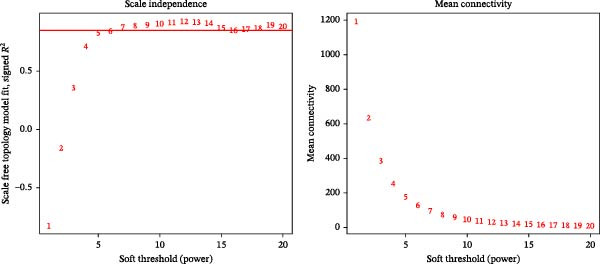
(B)

**Figure:**
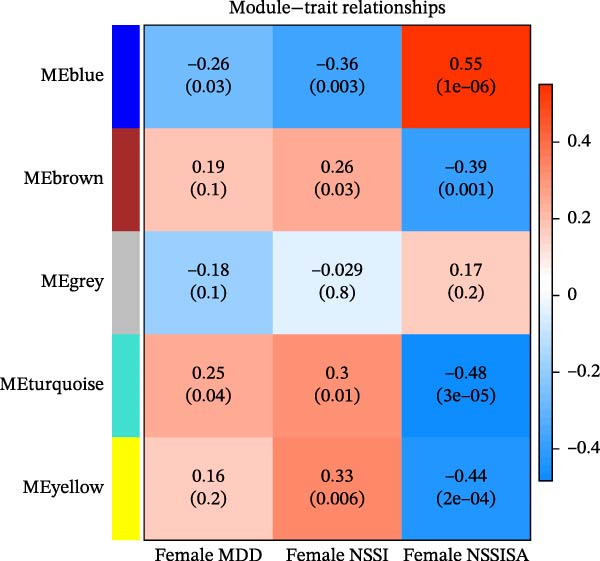
(C)

**Figure:**
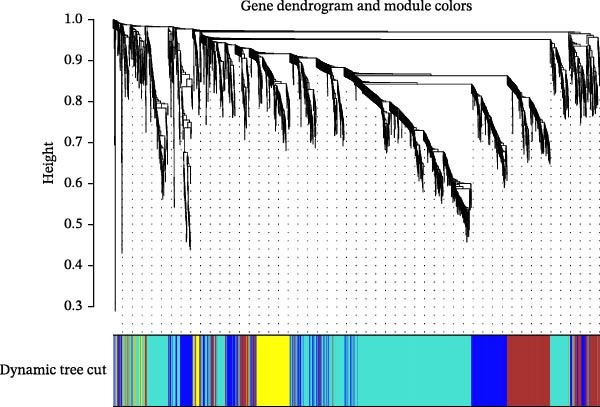
(D)

**Figure:**
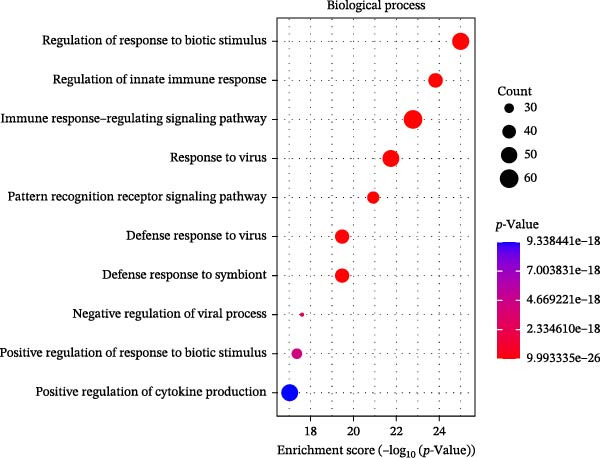
(E)

**Figure:**
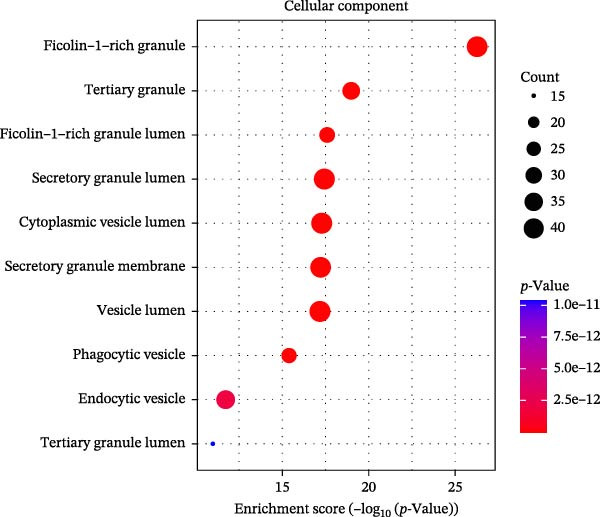
(F)

**Figure:**
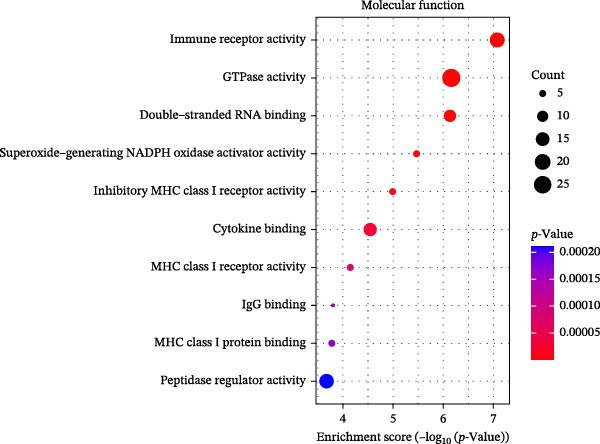
(G)

**Figure:**
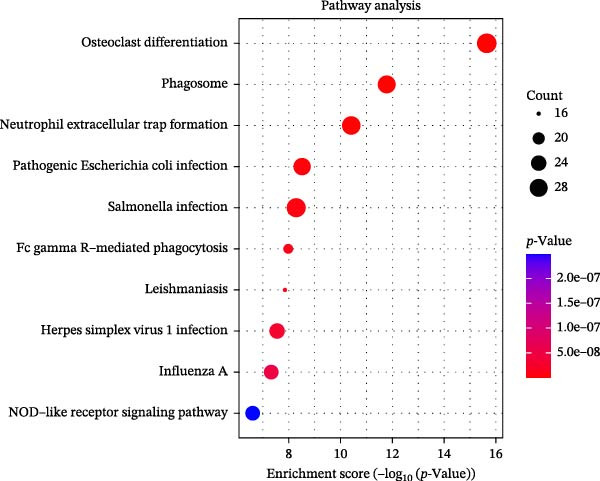
(H)

**Figure:**
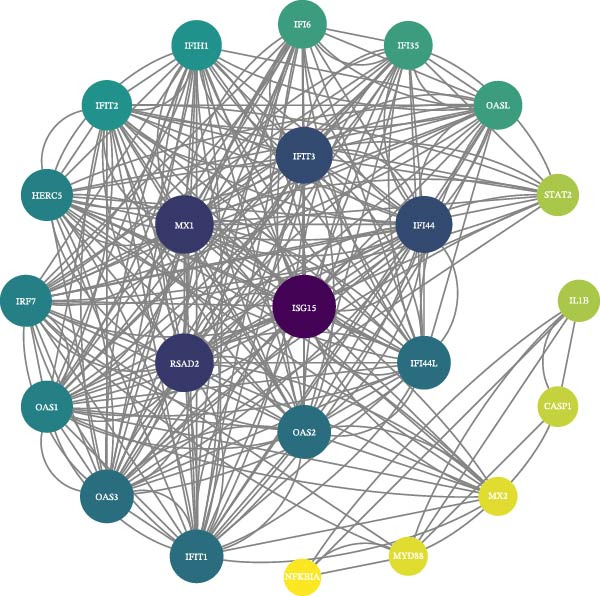
(I)

**Figure:**
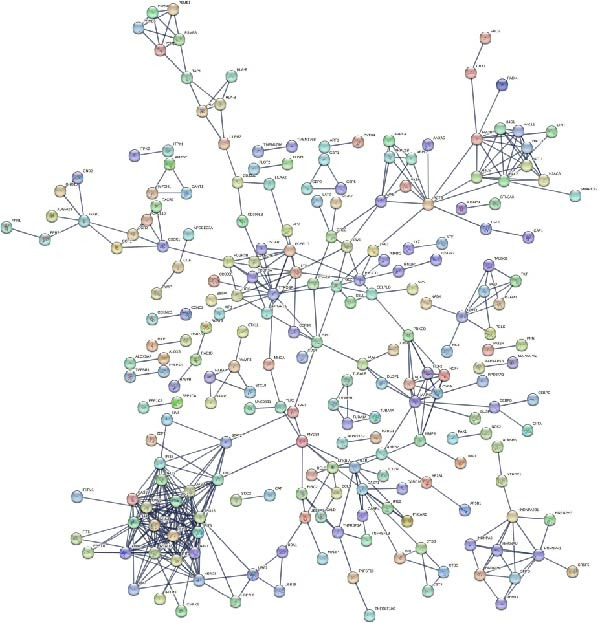
(J)

## 4. Discussion

This study used a whole‐blood transcriptomics analysis system to investigate the molecular mechanisms underlying the development of NSSI and SA in adolescents with MDD. First, the study identified significant differences in gene expression patterns: 1276 genes were differentially upregulated and 245 genes were differentially downregulated in the NSSISA group compared to the MDD group. Compared to the NSSI group, the NSSISA group exhibited 1658 upregulated genes and 408 downregulated genes. PPI network analysis of the DEGs identified two core hub genes: *NDUFS7* and *CXCL10*. *NDUFS7* serves as a core subunit of the mitochondrial respiratory chain complex I (CI), regulating NADH oxidation and proton transmembrane transport. It plays a critical role in maintaining neuronal energy metabolism (ATP production) and neurotransmitter homeostasis. *CXCL10* is a classic Th1‐type chemokine, primarily induced by interferons (such as IFN‐γ). It acts as a central mediator in immune inflammatory responses.

Using WGCNA and an integrated exploration of DEGs, we performed KEGG pathway and GO functional enrichment analyses on 62,657 genes. We found that these genes were significantly involved in multiple biological processes. This systematic approach provides a robust foundation for identifying functionally relevant gene modules in complex psychiatric disorders. Based on Cytoscape’s degree score, we identified 10 hub genes (*RSAD2*, *MX1*, *IFIT3*, *IFI44*, *IFI44L*, *OAS2*, *IFIT1*, *OAS3*, *OAS1*, and *IRF7*) that are closely associated with ISG15. These genes belong to the core network of ISGs and play central roles in innate immunity, especially in antiviral defense, as effectors and regulators. Notably, their relevance extends beyond infection to immune dysregulation commonly observed in psychiatric conditions. *ISG15* is a ubiquitin‐like protein and a key downstream factor in the type I interferon signaling pathway and the cytoplasmic DNA/RNA sensing pathway in response to viral infection. *ISG15* modifies target proteins (including viral and host proteins) through ISGylation, which precisely regulates their activity, localization, and stability. This inhibits viral replication [56, 57]. It is crucial to recognize that these molecular mechanisms also have profound implications for neuroimmune interactions. The ISG core gene set, which includes *ISG15*, can function as a “molecular marker” for evaluating the status of the type I interferon response. The observed synergistic effect is indicative of a type I interferon storm or sustained activation state within the body, accompanied by the complete activation of core antiviral mechanisms. From a clinical psychiatric perspective, such sustained immune activation may contribute to the pathophysiology of mood and behavioral disorders. This finding is of significant value in facilitating our understanding of immunopathological mechanisms, the development of targeted diagnostic tools, and the exploration of novel therapeutic interventions, including ISGylation inhibitors.

At the pathological mechanism level, *ISG15* has been found to be closely associated with depression and behavioral risks. This highlights the growing importance of immunopsychiatry in linking innate immunity to mental health. As demonstrated by a substantial body of research, the maternal immune activation (MIA) model has revealed a relationship between the expression of *ISG15* in the prefrontal cortex (PFC) of offspring and the subsequent induction of dendritic damage. This process is characterized by the inhibition of the Nedd4‐2/Rap2a pathway, which has been linked to the manifestation of depressive‐like behaviors. Notably, the inhibition of *ISG15* has been observed to result in a significant improvement in the symptoms associated with this condition [58]. These findings underscore ISG15 as a promising therapeutic target for immune‐related depressive phenotypes. Further studies revealed that chronic stress induced sustained high expression of *ISG15* in microglia, which promoted neuroinflammatory responses and abnormal synaptic pruning (via the complement pathway). This, in turn, led to the loss of synaptic marker *PSD95* in the PFC and social behavioral deficits. The knockout of the *IFNAR* gene has been demonstrated to effectively reduce *ISG15* expression in microglia, thereby confirming that the synaptic damage mediated by *ISG15* is directly associated with behavioral deficits [59]. This mechanistic insight bridges immune activation, glial dysfunction, and synaptic integrity in the context of behavioral regulation. ISG15 modifies synaptic proteins through ISGylation, impairing the executive control functions (such as impulse inhibition) of the PFC, which may significantly increase the risk of self‐injury. This offers a molecular framework for understanding how immune dysregulation may predispose individuals to impulsivity and self‐harm, frequently encountered in clinical psychiatric practice.

Another hub gene, *NDUFS7*, has been demonstrated to influence suicide risk through mitochondrial dysfunction pathways. This represents a crucial molecular link between cellular energy metabolism and psychiatric vulnerability. This gene encodes the structural core subunit (molecular weight 22–24 kD) of mitochondrial respiratory chain CI, which is involved in NADH oxidation, proton transmembrane transport, and energy production. The mutation in question has been demonstrated to induce CI dysfunction, thereby triggering a range of mitochondrial diseases, including Leigh syndrome. Patients frequently present with neuropsychiatric symptoms, including anxiety and cognitive impairment [60]. These clinical manifestations underscore the profound impact of metabolic integrity on brain function and emotional regulation. Mitochondrial CI dysfunction involving *NDUFS7* may exert an indirect influence on suicide risk through neuro‐metabolic mechanisms. This pathway elucidates how deficits in cellular energy production can converge with neural circuit dysfunction to elevate behavioral risk. Research has identified the presence of specific compounds in the blood of individuals diagnosed with depression and SI. These compounds, in some cases, have been linked to mitochondrial dysfunction. These compounds have the potential to serve as biomarkers, which could facilitate the classification of patients with treatment‐resistant depression and SI [61]. The emergence of such biomarkers is particularly promising for defining biologically homogeneous subgroups within the heterogeneous population of mood disorders, paving the way for personalized treatment strategies. As Zhang et al. [62] have indicated, there is a notable correlation between cytokine levels and depressive symptoms in patients diagnosed with depression. Mitochondrial dysfunction has been demonstrated to mediate changes in proinflammatory factor expression. This establishes a vicious cycle wherein metabolic impairment and immune activation mutually reinforce each other, exacerbating neuropsychiatric decline. In patients diagnosed with depression, mitochondrial dysfunction has been shown to lead to increased oxidative stress and disruption of calcium ion homeostasis. This, in turn, has been observed to affect the mood and behavior of patients with depression and to increase the risk of suicide [62]. Ultimately, this cascade of cellular dysfunction—from energy deficit and oxidative damage to neuroinflammation—compromises neuronal plasticity and survival, creating a neurobiological substrate conducive to impulsivity, hopelessness, and ultimately, suicidal behavior.

*CXCL10*, a pivotal molecule in the immune‐inflammatory axis, exhibits substantial clinical implications. Its central role underscores the growing recognition of immunopsychiatric mechanisms in mood disorders. Serum levels of the chemokine C–X–C motif ligand 10 (*CXCL10*) are significantly elevated in patients with first‐episode MDD (FE‐MDMD). This elevation is characterized by activation of the TNF signaling pathway and chemokine networks, making it a key biomarker of Th1‐polarized immune inflammation [63]. Its synthesis is directly driven by IFN‐γ, reflecting the intensity of the Th1 immune response. This places *CXCL10* within a well‐defined inflammatory cascade that is increasingly relevant to neuropsychiatric phenotypes. A multitude of studies have demonstrated that serum levels of the *CXCL10* are considerably elevated in patients diagnosed with familial exotropia‐myopia syndrome (FE‐MDMD) when compared to healthy control subjects. This finding suggests that the protein, known as *CXCL10*, is a significant biomarker of immune dysfunction in individuals diagnosed with frontotemporal dementia and myopathy. The consistency of this finding across studies strengthens its validity as a core inflammatory marker in affective illnesses. *CXCL10* is classified as a Th1‐type chemokine, which is a type of chemokine that typically plays a role in the immune system’s response to pathogens. The process is primarily induced by interferons (particularly IFN‐γ). The Th1 polarization (elevated Th1/Th2 ratio) and IL‐16‐driven activation of CD4^+^ T cells identified in studies provide upstream signaling cues for the production of *CXCL10*. Activation of Th1 cells secrete IFN‐γ, which further stimulates various cells, such as mononuclear/macrophage cells, astrocytes, and endothelial cells, to produce the chemokine. This detailed mechanism highlights how peripheral immune activation can directly influence central nervous system signaling and neuroinflammation. Consequently, elevated levels of the *CXCL10*, directly reflect the activation of the Th1 immune response and serve as an important effector molecule. Furthermore, the augmented neurotoxicity is predominantly fueled by *IL-16*, *TNF-α*, *TRAIL*, *IL-6*, *CCL2*, *CCL11*, *CXCL1*, *CXCL10*, *M-CSF*, and an array of signaling pathways. It is critical to recognize that *CXCL10* operates within a broader inflammatory network, collectively contributing to neuronal damage and synaptic dysfunction. This finding indicates that, in addition to its role in immune cell recruitment, *CXCR4* may also possess direct or indirect neurotoxic properties. In addition, a significant positive correlation between the severity of depression, anxiety, and suicidal behavior on the one hand, and the levels of the chemokine *CXCR3* on the other hand, has been demonstrated. This finding suggests that elevated levels of *CXCR3* are associated with more severe depressive and anxiety symptoms, and perhaps with an increased risk of suicide. This correlation positions *CXCL10* not only as a diagnostic marker but also as a potential predictor of clinical severity and complicating behaviors. This finding further substantiates the potential of *CXCR4* as a biomarker for disease severity and poor prognosis. Xu et al. [64] found that serum levels of the *CXCL10* were significantly higher in MDD patients with SI compared to those without SI. These findings suggest that the level of *CXCL10* in the serum could serve as a supplementary indicator for SI risk in inflammatory subtypes of MDD. This is of particular clinical value, as it offers a potential biological tool for stratifying suicide risk in a subset of patients with immune dysregulation. Research has indicated that levels of the chemokine IP‐10 in the cerebrospinal fluid of individuals who have attempted suicide are significantly lower than those in individuals who have not committed suicide. These findings suggest that IP‐10 may serve as a biomarker for long‐term suicide risk. While the present study concentrated on cerebrospinal fluid, it underscores the bidirectional regulatory function of *CXCR3* in suicidal behavior [65]. The discordant findings between peripheral and central levels remind us that the translation from blood to brain is not always linear and requires careful interpretation in the context of blood–brain barrier dynamics.

When interpreting these results, it is important to consider the study’s limitations. The sample size was limited and geographically concentrated, which may have reduced its statistical power and increased the risk of overlooking actual associations. This also limits the generalizability of the findings to adolescent girls from more diverse backgrounds. It is recommended that future studies employ larger, multicenter samples and utilize pre‐test power analysis in order to confirm and extend these results. The research’s controlled design offers valuable preliminary data and theoretical insight into the relationship between specific psychosocial factors and the mental health of adolescent girls.

This study also has methodological limitations beyond its sample size. First, while RNA‐seq offers high sensitivity, its results may be affected by technical biases, such as variations in reverse transcription efficiency and sequencing depth, potentially compromising the accuracy of low‐abundance transcripts. Second, findings depend on specific bioinformatics choices; different normalization or correction methods could alter results for genes of borderline significance. Functional enrichment analysis is constrained by existing database coverage and should be interpreted cautiously as indirect evidence. Finally, the cross‐sectional design identifies correlations in gene expression but cannot establish causality or temporal dynamics relative to behavior. These limitations call for cautious interpretation and further validation through independent replication and longitudinal or experimental designs.

A key limitation of this study is its reliance on peripheral blood gene expression profiling, which does not directly examine corresponding changes within the brain. While peripheral blood provides accessible systemic data, it is important to note that findings cannot be equated to molecular events in the brain due to the presence of the blood–brain barrier and tissue‐specific expression. This limitation, therefore, results in a restriction to the mechanistic interpretation of the biomarkers. Multimodal brain imaging studies have been proposed as a means of addressing this gap. For instance, a multicentre study established a correlation between impaired network topology in regions such as the prefrontal and anterior cingulate cortices in depressed patients with SI and gene expression patterns involved in cellular metabolism and synaptic signaling [66]. This finding is consistent with the conclusions of reviews indicating that SI involves abnormalities in frontocortical limbic circuits related to emotion regulation and cognitive control [67]. It is noteworthy that functional abnormalities in the dorsal anterior cingulate cortex, a region critical for cognitive control, have been consistently documented in studies of suicidal behavior [68]. This highlights its pivotal role in regulating negative emotions and impulsivity. It is recommended that future research adopts integrated designs that combine multimodal neuroimaging with peripheral multiomics analysis in the same cohort. This would facilitate direct validation of the links between peripheral markers and specific brain circuit abnormalities, thus helping to construct a comprehensive pathological model spanning molecular expression, brain networks, and clinical phenotypes. The importance of such work lies in its potential to advance the field of precision assessment and intervention for suicide risk in depression.

While this study provides a descriptive account of gender differences in NSSI and SA, its design lacked deep biological measures, precluding analysis of underlying mechanisms. Future prospective research should systematically collect biological variables, such as through menstrual cycle tracking or direct hormone assays, to dynamically examine how hormonal fluctuations relate to impulsivity and emotional regulation. A particular focus should be on estrogen’s regulatory role in serotonergic and GABAergic neuroplasticity. Estrogen may enhance serotonin synthesis and modulate receptor expression, potentially explaining higher rates of mood disorders in women during hormonal transitions [62, 69, 70]. Research should also explore the link between androgens and impulsive aggression, noting testosterone’s complex influence on brain regions like the amygdala and PFC within social contexts [71]. Future studies should integrate genetic data on hormone‐related genes with endogenous hormone levels and environmental stressors to clarify how neuroendocrine patterns shape behavioral risk. A comprehensive investigation is needed to elucidate how sex hormones regulate immune processes and influence prefrontal‐limbic circuitry [72]. To disentangle the effects of biological sex from social gender, future studies could include other gender individuals. Comparing NSSI/SA risk and neurobiological markers before and after hormone therapy would provide stronger evidence for the direct effects of hormones.

## 5. Conclusions

This study systematically reveals the multidimensional heterogeneity of the high‐risk phenotype characterized by NSSI and SA in female adolescents with MDD at both clinical and transcriptomic levels, and provides preliminary elucidations of the underlying key molecular mechanisms. The findings indicate that *NDUFS7* and *CXCL10* function as core hub genes, exhibiting strong predictive value for the MDD with NSSI + SA phenotype. In addition, immune gene modules that are closely associated with the severity of self‐injury, such as MEblue (identified through WGCNA analysis), collectively indicate the pivotal role of neuroimmune activation and interferon signaling pathways in the development of this behavioral phenotype. The present findings indicate that interventions targeting key genes, such as *NDUFS7* and *CXCL10*, and their associated neuroimmune pathways, may provide a novel therapeutic approach for the precise diagnosis and treatment of this specific subtype of MDD with NSSI/SA in female adolescents.

## Author Contributions

Zimo Zhou and Lin Tian conceived the study. Zimo Zhou, Lin Tian, Jiajun Yin, and Shuai Wang wrote the protocol. Zimo Zhou, Zhenru Guo, Lianlian Yang, Yu Xia, Yuanyuan Yang, and Zhangyan Shan performed the analyses. Zimo Zhou wrote the first draft.

## Funding

This research was supported by the Talent Support Programs of Wuxi Health Commission (Grants BJ2023085, M202167, and M202358), the Wuxi Taihu Talent Project (Grant WXTTP2021), the Medical Key Discipline Program of Wuxi Health Commission (Grant FZXK2021012), and the Science and Technology Development Fund of Wuxi City (Grant Y20252221).

## Disclosure

All claims expressed in this article are solely those of the authors and do not necessarily represent the views of their affiliated organizations, the publisher, the editors, or the reviewers. Any product that may be evaluated in this article, or any claim that may be made by its manufacturer, is not guaranteed or endorsed by the publisher. All authors have read, provided comments on, and approved the final version of the manuscript.

## Conflicts of Interest

The authors declare no conflicts of interest.

## Data Availability

The data that support the findings of this study are available upon request from the corresponding author. The data are not publicly available due to privacy or ethical restrictions.
